# Ryanodine Receptor-Mediated Calcium Release Has a Key Role in Hippocampal LTD Induction

**DOI:** 10.3389/fncel.2018.00403

**Published:** 2018-11-06

**Authors:** Alejandra Arias-Cavieres, Genaro C. Barrientos, Gina Sánchez, Claudio Elgueta, Pablo Muñoz, Cecilia Hidalgo

**Affiliations:** ^1^Biomedical Neuroscience Institute (BNI), Faculty of Medicine, Universidad de Chile, Santiago, Chile; ^2^Physiology and Biophysics Program, Institute of Biomedical Sciences, Faculty of Medicine, Universidad de Chile, Santiago, Chile; ^3^Pathophysiology Program, Institute of Biomedical Sciences, Faculty of Medicine, Universidad de Chile, Santiago, Chile; ^4^Systemic and Cellular Neurophysiology, Physiology Institute I, Medical Faculty, University of Freiburg, Freiburg, Germany; ^5^Pathology and Physiology Department, Medical School, Faculty of Medicine, Universidad de Valparaíso, Valparaíso, Chile; ^6^Department of Neuroscience and Center of Molecular Studies of the Cell, Faculty of Medicine, Universidad de Chile, Santiago, Chile

**Keywords:** calcium signals, ryanodine, caffeine, basal synaptic transmission, low-frequency stimulation

## Abstract

The induction of both long-term potentiation (LTP) and long-term depression (LTD) of synaptic transmission entails pre- and postsynaptic Ca^2+^ signals, which represent transient increments in cytoplasmic free Ca^2+^ concentration. In diverse synapse types, Ca^2+^ release from intracellular stores contributes to amplify the Ca^2+^ signals initially generated by activation of neuronal Ca^2+^ entry pathways. Here, we used hippocampal slices from young male rats to evaluate whether pharmacological activation or inhibition of Ca^2+^ release from the endoplasmic reticulum (ER) mediated by ryanodine receptor (RyR) channels modifies LTD induction at Schaffer collateral-CA1 synapses. Pre-incubation of slices with ryanodine (1 μM, 1 h) or caffeine (1 mM, 30 min) to promote RyR-mediated Ca^2+^ release facilitated LTD induction by low frequency stimulation (LFS), but did not affect the amplitude of synaptic transmission, the profiles of field excitatory postsynaptic potentials (fEPSP) or the paired-pulse (PP) responses. Conversely, treatment with inhibitory ryanodine (20 μM, 1 h) to suppress RyR-mediated Ca^2+^ release prevented LTD induction, but did not affect baseline synaptic transmission or PP responses. Previous literature reports indicate that LTD induction requires presynaptic CaMKII activity. We found that 1 h after applying the LTD induction protocol, slices displayed a significant increase in CaMKII phosphorylation relative to the levels exhibited by un-stimulated (naïve) slices. In addition, LTD induction (1 h) enhanced the phosphorylation of the presynaptic protein Synapsin I at a CaMKII-dependent phosphorylation site, indicating that LTD induction stimulates presynaptic CaMKII activity. Pre-incubation of slices with 20 μM ryanodine abolished the increased CaMKII and Synapsin I phosphorylation induced by LTD, whereas naïve slices pre-incubated with inhibitory ryanodine displayed similar CaMKII and Synapsin I phosphorylation levels as naïve control slices. We posit that inhibitory ryanodine suppressed LTD-induced presynaptic CaMKII activity, as evidenced by the suppression of Synapsin I phosphorylation induced by LTD. Accordingly, we propose that presynaptic RyR-mediated Ca^2+^ signals contribute to LTD induction at Schaffer collateral-CA1 synapses.

## Introduction

Evidence gathered in the last three decades supports long-term potentiation (LTP) and long-term depression (LTD) as likely cellular correlates of associative learning and long-term memory (Dudek and Bear, 1992; Bliss and Collingridge, 1993; Bear and Abraham, 1996; Malenka and Bear, 2004; Whitlock et al., 2006; Raymond, 2007; Citri and Malenka, 2008; Lynch et al., 2014; Nabavi et al., 2014). Both LTP and LTD require increments in postsynaptic intracellular Ca^2+^ concentration (Mulkey and Malenka, 1992), which occur initially by means of Ca^2+^ influx through *N*-methyl-D-aspartate (NMDA) receptors or voltage-gated Ca^2+^ channels (Dudek and Bear, 1992; Mulkey and Malenka, 1992; Citri and Malenka, 2008). In addition, LTD induction entails presynaptic CaMKII activation, whereas postsynaptic CaMKII stimulation is required to evoke LTP (Stanton and Gage, 1996; Margrie et al., 1998).

Substantial evidence implicates ensuing amplification of Ca^2+^ entry signals by means of Ca^2+^ release from intracellular stores mediated by the two types of Ca^2+^ release channels present in the endoplasmic reticulum (ER), the inositol 1,4,5-trisphosphate (IP_3_) receptor (IP_3_R) and the ryanodine receptor (RyR) channels (Berridge, 1998, 2002). The IP_3_R channels, which play an important role in neuronal function, reside predominantly in the cerebellum (Hisatsune and Mikoshiba, 2017) and the hippocampus (Sharp et al., 1993; Hertle and Yeckel, 2007). The RyR channels, which are widely expressed in the brain (Furuichi et al., 1994; Zhao et al., 2000), play central roles in synaptic plasticity (Reyes and Stanton, 1996; Lu and Hawkins, 2002; Raymond and Redman, 2002; Mellentin et al., 2007; Grigoryan et al., 2012). In addition, RyR channel activation facilitates learning and memory formation whereas inhibition of RyR activity or expression impairs these processes (Zhao et al., 2000; Edwards and Rickard, 2006; Galeotti et al., 2008; Adasme et al., 2011; Hidalgo and Arias-Cavieres, 2016; More et al., 2018).

As implied by its name, RyR channels bind selectively and with high affinity the plant alkaloid ryanodine (Jenden and Fairhurst, 1969). Ryanodine at low concentrations (≤1 μM) activates RyR channels, while higher ryanodine concentrations (≥10 μM) inhibit RyR single channel activity (Meissner, 1986; Bull et al., 1989; Mackrill, 2010). We have reported previously that inhibitory ryanodine concentrations (≥10 μM) suppress RyR-mediated Ca^2+^ release induced by high-frequency stimulation of primary hippocampal neurons (Riquelme et al., 2011) but do not affect ER calcium content (Adasme et al., 2015). Considering that ryanodine binds predominantly to open RyR channels, full inhibition of RyR-mediated Ca^2+^ release requires prolonged incubation (≥1 h) with inhibitory ryanodine. In addition, caffeine at mM concentration also promotes full RyR channel activation (McPherson et al., 1991); however, whereas ryanodine only targets RyR channels, caffeine also inhibits cyclic nucleotide phosphodiesterases (Sutherland and Rall, 1958) and IP_3_R channel activity (Bezprozvanny et al., 1994), and antagonizes adenosine receptors (Rall, 1982).

Previous reports have indicated that RyR channels play important roles in the LTP and LTD responses recorded in different hippocampal regions (Baker et al., 2013; Paula-Lima et al., 2014). In Schaffer collateral-CA1 synapses, RyR channel inhibition prevents LTP induction or maintenance in CA3–CA1 synapses, depending on the type of stimulation protocol used, theta-burst stimulation (TBS) or high-frequency stimulation (Lu and Hawkins, 2002; Raymond and Redman, 2002; Mellentin et al., 2007; Grigoryan et al., 2012). Inhibition of RyR channels prevents LTD induction in dentate gyrus (DG) synapses (Wang et al., 1997), while in CA3–CA3 synapses, presynaptic ryanodine-sensitive Ca^2+^ stores are required for NMDA receptor (NMDAR)-dependent LTD induction (Unni et al., 2004). Depletion of intracellular Ca^2+^ stores does not affect baseline synaptic transmission in CA3–CA1 synapses but blocks LTD induction in young rats (Reyes and Stanton, 1996). Moreover, based on the findings that bath application of inhibitory ryanodine (10 μM) blocks LTD induction whereas filling CA1 pyramidal neurons with ryanodine (2 μM to 5 mM) does not, the authors proposed that LTD induction requires Ca^2+^ release from presynaptic ryanodine-sensitive Ca^2+^ stores and from postsynaptic (presumably IP_3_-gated) stores (Reyes and Stanton, 1996). In contrast, a subsequent report proposed that postsynaptic calcium stores are critical for LTD induction (Nakano et al., 2004). In CA3–CA1 synapses, LTD induction at homo- and heterosynaptic sites requires functional RyR and IP_3_R channels, respectively (Nishiyama et al., 2000). Furthermore, knockout mice for the type-3 RyR (RyR3) isoform do not display LTD in CA3–CA1 synapses (Futatsugi et al., 1999), albeit the synaptic location of RyR3 channels was not established. In addition, RyR channels contribute to both the defective synaptic plasticity (Kumar and Foster, 2005) and the neuronal Ca^2+^ dysregulation displayed by aged animals (Gant et al., 2006, 2015; Bodhinathan et al., 2010). In particular, we recently reported that aged rats display increased RyR2 and RyR3 protein levels and significantly enhanced LTD in CA3–CA1 synapses (Arias-Cavieres et al., 2017). These combined results indicate that RyR channels play key roles in LTD induction in Schaffer collateral-CA1 synapses.

To examine if modifying RyR channel activity affects circuit excitability in hippocampal slices from young male rats (28–35 days old), we studied in Schaffer collateral-CA1 synapses the effects of low (1 μM) or high (20 μM) concentrations of ryanodine or of 1 mM caffeine on input/output (I/O) responses. We evaluated as well the effects of ryanodine and caffeine on fast presynaptic transmitter release dynamics by studying CA1 paired-pulse (PP) responses after stimulation of Schaffer collateral fibers, and assessed the effects of both drugs on LTD induction by low frequency stimulation (LFS) of hippocampal slices. We also examined in control slices and in slices treated with 20 μM ryanodine if LTD induction modified the phosphorylation levels of CaMKII, a protein expressed in pre- and postsynaptic terminals that has a key role in LTD induction (Stanton and Gage, 1996; Margrie et al., 1998; Coultrap and Bayer, 2012). To evaluate presynaptic CaMKII activity, we measured the phosphorylation of the presynaptic protein Synapsin I at a site that undergoes specific Ca^2+^-dependent phosphorylation by CaMKII (Cesca et al., 2010).

## Materials and Methods

### Materials

All reagents used were of analytical grade. Stock solutions of ryanodine (Tocris, Bristol, UK) and caffeine (Sigma, St. Louis, MO, USA) were dissolved in water and stored in aliquots at −20°C before thawing and dilution to their final concentrations in artificial cerebrospinal fluid (ACSF) solution (in mM: 124 NaCl, 5 KCl, 1 MgCl_2_, 2 CaCl_2_, 1.25 NaH_2_PO_4_, 26 NaHCO_3_, pH 7.4, 10 glucose). The ACSF solution was oxygenated with a mixture of 95% O_2_/5% CO_2_. Antibodies: anti-CamKII (pan) (D11A10) rabbit monoclonal antibody (#4436, 1:3,000) was from Cell Signaling Technology (Danvers, MA, USA). Phospho CaMKII (phospho T286) rabbit polyclonal antibody (ab32678, 1:5,000), anti-synapsin I rabbit polyclonal antibody (ab64581, 1:2,500) and anti-synapsin I (phospho S603) rabbit polyclonal antibody (ab13879, 1:1,500) were from Abcam (Cambridge, MA, USA).

### Animals

Young (4–5 weeks) male *Sprague Dawley* rats were obtained from the Universidad de Chile animal facility. Food and water were provided *ad libitum*. Lights were maintained on a 12–12 light/dark cycle. All experiments were carried out following the guidelines provided by National Institute of Health (USA) and the regulations for the Care and Use of Animals for Scientific Purposes; the Bioethics Committee, F. Medicine, Universidad de Chile, approved all protocols used in this work.

### Hippocampal Slice Preparation

Animals were sacrificed by decapitation under halothane anesthesia and their brains were quickly removed. The hippocampal tissue was removed, dissected and immersed in cold dissection buffer (in mM: 212.7 sucrose, 10 glucose, 5 KCl, 1.25 NaH_2_PO_4_, 2 MgCl_2_, 1 CaCl_2_, 26 NaHCO_3_, pH 7.4) and cut into 400 μm transversal slices with a VT 1000 S vibratome (Leica, Wetzlar, Germany). Hippocampal slices were transferred to an immersion storage chamber and were kept at room temperature for 1 h in ACSF solution, bubbled with 95% O_2_/5% CO_2_.

### Electrophysiology

For field recordings slices were perfused with ACSF bubbled with 95% O_2_/5% CO_2_ (30 ± 2°C) at a rate of 2 ml/min. Synaptic transmission at the Schaffer collaterals-CA1 synapse was evoked by square current pulses (0.2 ms) delivered with a concentric bipolar stimulating electrode (FHC Inc., Bowdoinham, ME, USA) placed at the Schaeffer collateral–commissural fibers (Arias-Cavieres et al., 2017); field excitatory postsynaptic potentials (fEPSP) were recorded with ACSF-filled glass microelectrodes (2–3 MΩ) placed into the CA1 *stratum radiatum* region. Pulses of 50, 100, 150, 200 or 250 microamperes were applied to generate I/O response curves using a constant current stimulator (AM system, Washington, DC, USA). Signals were amplified and filtered at 10 kHz using a differential amplifier (AM system, Washington, DC, USA); signals were digitized using a national instruments board (NI PCI6221, Austin, TX, USA) and recorded using custom-made routines written in Igor Pro (WaveMetrics Inc., Lake Oswego, OR, USA). The experiment was discarded if a population spike was detected (Andersen et al., 1980).

PP experiments were performed to evaluate synaptic release dynamics. PPs were evoked every 15 s with time delays ranging from 20 ms to 640 ms. Plasticity experiments were performed after synaptic transmission and PP determinations. Pulses were delivered every 15 s, using stimulation intensities that evoked half-maximal fEPSP amplitudes. After collecting baseline responses for 15 min, an LFS protocol (1 Hz/900 pulses) was applied and fEPSPs were registered for 60 min to test for LTD induction.

### Data Analysis of Electrophysiological Records

Data analysis was done using custom-made software written in Igor Pro (WaveMetrics Inc., Lake Oswego, OR, USA). Fiber volley (FV; Andersen et al., 1980) and fEPSP amplitudes were measured as the peak negative response from baseline. Rise times were measured as the time elapsed between reaching 20–80 percent of the peak fEPSP amplitude, and half-width time as the width of fEPSP traces at half amplitude. Decay constant (tau) vales were determined by fitting a single exponential function to the after-peak fEPSP waveform. Results from PP experiments are presented as fEPSP slope ratios. Plasticity experiments are presented as percent change of the initial fEPSP slopes.

### Western Blot Analysis

Two-three hippocampal slices (400 μm each) from each rat were pooled and extracts were prepared as described (Arias-Cavieres et al., 2017). Proteins were resolved by SDS-PAGE using 3.5%–8% Tris-acetate gels (RyR2) and 10% gels (Synapsin I and CaMKII). Proteins were transferred to polyvinylidenedifluoride (PVDF) membranes, and probed with CaMKII, phospho-CaMKII, Synapsin I and phospho-Synapsin I antibodies. Image acquisition was performed by means of the Chemidoc™ MP System (Bio-Rad laboratories, Hercules, CA, USA); the ImageJ Lab software was used for band density analysis.

### Immunohistochemistry

Free-floating sections for immunofluorescence were prepared as previously described (Muñoz et al., 2016). Naïve slices or slices exposed to LTD induction protocols (plus or minus 20 μM ryanodine in both cases), were fixed for 30 min in 4% paraformaldehyde/4% sucrose and then placed in 30% sucrose (w/v). The fixed slices were washed three times with phosphate-based saline buffer, embedded in medium for frozen tissue specimens and sectioned at −20°C with a cryostat to generate sections of 25 μm width. Free-floating sections were incubated overnight at 4°C with permeabilization/blocking buffer (0.7% Triton X-100, 0.1% sodium borohydride, 10% goat serum), and were subsequently incubated for 12 h at 4°C with phospho-CaMKII rabbit polyclonal antibody (phospho T286, ab32678, 1:500), or with phospho-Synapsin I rabbit polyclonal antibody (phospho S603, ab13879, 1:500). Both antibodies were from Abcam (Cambridge, MA, USA). After this incubation period, washed sections were incubated for 2 h with donkey-anti-rabbit Alexa Fluor 546 antibody (1:500, Fisher Scientific, Waltham, MA, USA). Nuclei were visualized with Hoechst 33342 (1:1,000, Fisher Scientific, Waltham, MA, USA). Images were acquired in a confocal microscope (Nikon Eclipse C180i, Melville, NY, USA).

### Statistics

Statistical analysis was performed using the GraphPad Software (San Diego, CA, USA), as detailed in figure legends. All values represent Mean ± SE. For independent data sets, statistical differences between two mean values were assessed by two-tailed unpaired Student’s *t*-test provided values presented normal distribution, determined by the Shapiro-Wilk test. The nonparametric Mann-Whitney U test was used if values did not present normal distribution or the sample number was ≤ 5. Differences were considered statistically significant at *p* < 0.05.

## Results

### Effects of Ryanodine and Caffeine on Input-Output Responses

**Figure 1:**
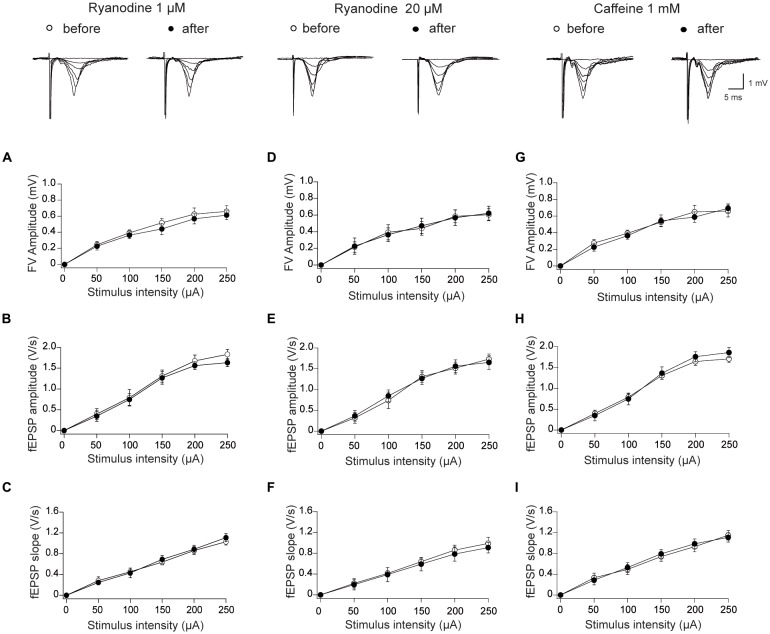
Effects of stimulatory and inhibitory ryanodine concentrations and of caffeine on basal synaptic transmission in the CA3–CA1 hippocampal synapse. Left panels: slices were treated with 1 μM ryanodine; representative field excitatory postsynaptic potential (fEPSP) traces registered before and 15 min after ryanodine addition are illustrated on top of the panels. **(A)** Fiber volley (FV) amplitude vs. stimulus intensity; **(B)** amplitude vs. stimulus intensity; **(C)** fEPSP slopes vs. stimulus intensity. Central panels: slices were treated with 20 μM ryanodine; representative fEPSP traces registered before and 60 min after ryanodine addition are illustrated on top. **(D)** FV amplitude vs. stimulus intensity; **(E)** fEPSP amplitude vs. stimulus intensity; **(F)** fEPSP slopes vs. stimulus intensity. Right Panels: slices were treated with 1 mM caffeine; representative fEPSP traces registered before and 15 min after caffeine addition are illustrated on top. **(G)** FV amplitude vs. stimulus intensity; **(H)** fEPSP amplitude vs. stimulus intensity; **(I)** fEPSP slopes vs. stimulus intensity. Values represent Mean ± SEM; (12, 3). The first number in parentheses indicates the number of hippocampal slices and the second the number of animals used. Statistical significance of values was assessed by Mann-Whitney test (*p* > 0.05 in all cases).

First, we explored if activating (1 μM) or inhibitory (20 μM) concentrations of ryanodine, as well as 1 mM caffeine, influenced signaling at the Schaffer collateral-CA1 synapses. To this aim, I/O curves were generated from recordings collected after 15 min of perfusion with control ACSF or with ACSF containing 1 μM ryanodine. At all tested stimulus intensities, 1 μM ryanodine had no effect on FV amplitudes (Figure 1A), fEPSP amplitudes (Figure 1B) or fEPSP slopes (Figure 1C). We evaluated next these three parameters in slices perfused for 60 min with 20 μM ryanodine. Treatment with inhibitory ryanodine did not affect FV amplitudes (Figure 1D), fEPSP amplitudes (Figure 1E) and fEPSP slopes (Figure 1F). Consistent with the lack of effect of stimulatory ryanodine, RyR activation by application of 1 mM caffeine did not affect FV amplitudes (Figure 1G), fEPSP amplitudes (Figure 1H) and fEPSP slopes (Figure 1I).

**Figure 2:**
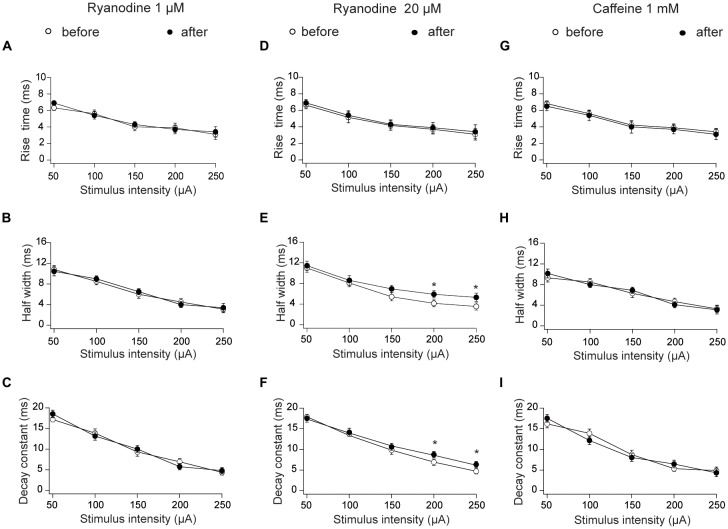
Effects of stimulatory and inhibitory ryanodine concentrations and of caffeine on fEPSP rise times, half-widths and decay constants tau measured in CA3–CA1 hippocampal synapses. Left panels: slices were treated with 1 μM ryanodine. **(A)** fEPSP rise times vs. stimulus intensity. **(B)** fEPSP half-widths vs. stimulus intensity; **(C)** fEPSP decay constants (tau) vs. stimulus intensity. Central panels: slices were treated with 20 μM ryanodine for 60 min. **(D)** fEPSP rise times vs. stimulus intensity; **(E)** fEPSP half-widths vs. stimulus intensity; **(F)** fEPSP decay constants (tau) vs. stimulus intensity. Right panels: slices were treated for 15 min with 1 mM caffeine. **(G)** fEPSP rise times vs. stimulus intensity; **(H)** fEPSP half-widths vs. stimulus intensity; **(I)** fEPSP decay constants (tau) vs. stimulus intensity. Values represent Mean ± SE; (12, 3). The first number in parentheses indicates the number of hippocampal slices and the second the number of animals used. Statistical significance of values was assessed Mann-Whitney test (*p < 0.05).

We also tested the effects of ryanodine and caffeine on additional fEPSP parameters, and found that 1 μM stimulatory ryanodine did not affect fEPSP rise times (Figure 2A), half-widths (Figure 2B) or decay rates (Figure 2C). Treatment with inhibitory ryanodine (20 μM) did not affect fEPSP rise times (Figure 2D) but caused a modest increase in half-widths (Figure 2E) and decay constant tau values (Figure 2F) displayed by fEPSPs evoked by strong stimulation (200 and 250 μA). As observed following treatment with stimulatory ryanodine, RyR activation by application of 1 mM caffeine had no effect on fEPSP rise times (Figure 2G), half-widths (Figure 2H) or decay rates (Figure 2I).

Based on these combined results, we conclude that RyR activation with ryanodine or caffeine does not modify the electrical signals recorded during basal synaptic transmission, and that RyR inhibition does not affect fEPSP properties in the stimulation range < 200 μA, a range that was used in all subsequent experiments.

### Activation or Inhibition of RyR Channels Does Not Affect Paired-Pulse Responses

**Figure 3:**
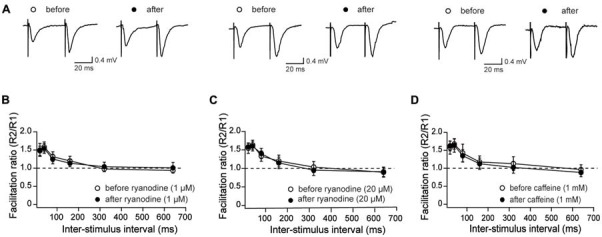
Effects of stimulatory and inhibitory ryanodine concentrations and of caffeine on paired-pulse (PP) responses measured in CA3–CA1 hippocampal synapses. **(A)** Representative fEPSP traces showing PP responses before and after addition of 1 μM ryanodine (left panels), 20 μM ryanodine (center panels) and 1 mM caffeine (right panels). **(B)** Effects of 1 μM ryanodine applied for 15 min on PP facilitation. The graph illustrates the facilitation ratio vs. inter-stimulus intervals. **(D)** Effects of 1 mM caffeine applied for 15 min on PP facilitation. The graph illustrates the facilitation ratio vs. inter-stimulus intervals. **(C)** Effects of 20 μM ryanodine applied for 60 min on PP facilitation. The graph illustrates the facilitation ratio vs. inter-stimulus intervals. Values represent Mean ± SE; (13, 4) for ryanodine-treated slices; (12, 3) for caffeine-treated slices and (16, 4) for the control. The first number in parentheses indicates the number of hippocampal slices and the second the number of animals used.

To analyze further if RyR-mediated Ca^2+^ release modifies the PP response, we evaluated this response in control conditions and after treatment of slices with stimulatory or inhibitory ryanodine concentrations, or with caffeine (Figure 3A). Treatment with 1 μM ryanodine (Figure 3B) or 1 mM caffeine (Figure 3D) to promote RyR channel activation did not affect fEPSP slope ratios, independent of the delay between pulses. Likewise, perfusion with 20 μM ryanodine did not affect the PP responses (Figure 3C). Hence, we suggest that RyR inhibition does not affect the generation of presynaptic local Ca^2+^ signals involved in this fast response.

### Modulation of LTD Induction by Ryanodine and Caffeine

**Figure 4:**
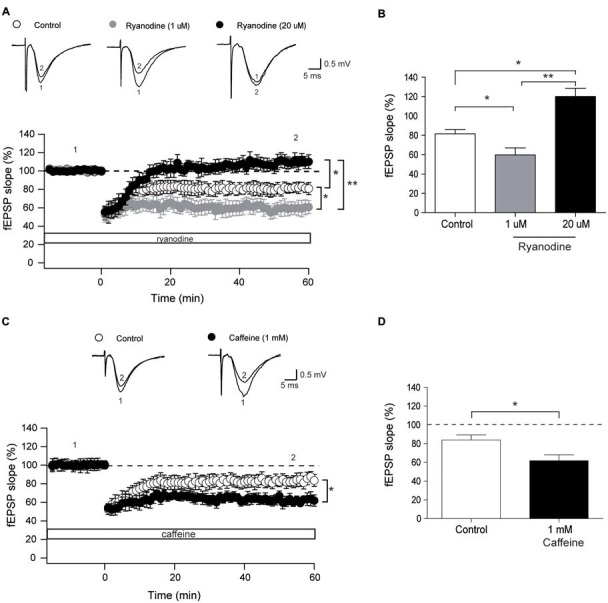
Stimulatory and inhibitory ryanodine concentrations and caffeine modify the long-term depression (LTD) response. **(A)** Time course of fEPSP slopes recorded (CA3–CA1) before and after application of the low frequency stimulation (LFS) protocol to control hippocampal slices (14, 4) or to slices treated with 1 μM ryanodine (14, 3) or 20 μM ryanodine (13, 4). Representative fEPSP traces recorded 1–5 min before (trace 1) and 60 min after applying the LFS protocol (trace 2) to control slices, or recorded in slices treated with 1 μM or 20 μM ryanodine are shown on top of the graph. Open symbols: control slices; gray symbols: slices treated with 1 μM ryanodine; black symbols: slices treated with 20 μM ryanodine. **(B)** Average magnitudes of fEPSP slopes recorded during the last 10 min after stimulation. **(C)** Time course of fEPSP slopes recorded (CA3–CA1) before and after application of the LFS protocol to control hippocampal slices (15, 3) or to slices treated with 1 mM caffeine (12, 4). The first number in parentheses indicates the number of hippocampal slices and the second the number of animals used. Representative fEPSP traces recorded 1–5 min before (trace 1) and 60 min (trace 2) after applying the LFS protocol to control slices and to slices treated with 1 mM caffeine are shown on top of the graph. Open symbols: control slices; black symbols: slices treated with 1 mM caffeine. **(D)** Average magnitudes of fEPSP slopes recorded during the last 10 min after stimulation of control or caffeine-treated slices. Values represent Mean ± SE. Statistical significance of values was assessed by Mann-Whitney test (*p < 0.05; **p < 0.01).

We evaluated next whether activation or inhibition of RyR channels affects LTD induction by LFS at 1 Hz. To this aim, we tested first the effects of incubating hippocampal slices with stimulatory (1 μM) ryanodine for 15 min before application of the LFS protocol, and found that this treatment significantly enhanced LTD induction (Figures 4A,B). Relative to the initial slope values, defined as 100%, 60 min after application of the LFS protocol control slices displayed fEPSP slopes (% values) of 81.4 ± 4.8, whereas slices treated with 1 μM ryanodine exhibited fEPSP slopes (% values) of 59.7 ± 7.2. Consistently, pre-incubation with 1 mM caffeine to activate RyR channels also enhanced LTD induction to the same extent as stimulatory ryanodine concentrations (Figures 4C,D). Caffeine-treated slices displayed fEPSP slope values (in %), measured 60 min after LFS, of 61.8 ± 6, which were significantly lower than the % values of 83.8 ± 5.4 displayed by control slices.

Incubation of hippocampal slices for 60 min with 20 μM ryanodine to abolish RyR channel activity did not affect basal transmission but completely prevented LTD induction (Figures 4A,B) with % fEPSP values of 120.1 ± 7.4, measured 60 min after the application of the LFS protocol.

### LTD Increases the Phosphorylation Levels of CaMKII and Synapsin I

**Figure 5:**
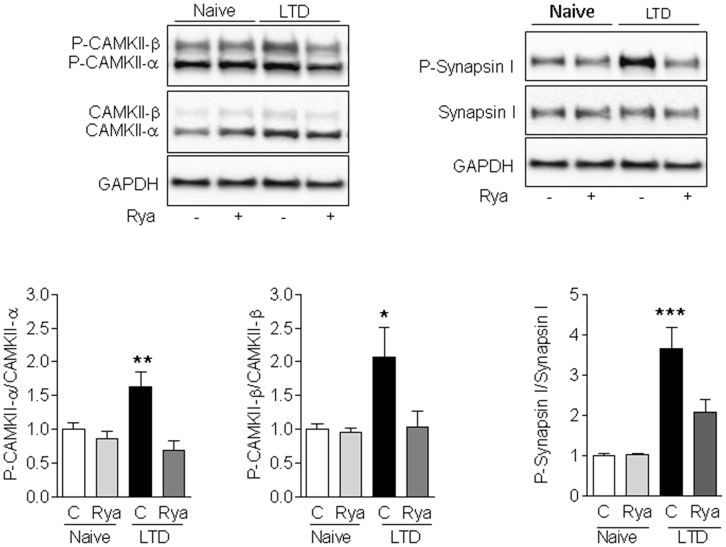
LTD-induced phosphorylation of CaMKII and Synapsin I. Induction of LTD for 60 min in control (C) slices caused a significant increase in the phosphorylation levels of Synapsin I (left panels), CaMKII-α (center panels) and CaMKII-β (right panels) relative to the levels displayed by unstimulated slices (naïve). Slices pre-incubated for 1 h with 20 μM ryanodine (Rya) before applying the LTD induction protocol displayed significantly lower increments in the phosphorylation levels of Synapsin I and CaMKII-α, whereas the phosphorylation levels of CaMKII-β were not significantly different from the levels displayed by unstimulated slices. Values represent Mean ± SE (*n* = 3). Statistical analysis was performed with one-way ANOVA, followed by Tukey’s *post hoc* test. *p < 0.05 vs. naïve; **p < 0.01 vs. naïve; ***p < 0.001 vs. naïve.

We evaluated next in slices if LTD-inducing protocols modified the Ca^2+^-dependent phosphorylation of CaMKII and of the presynaptic protein Synapsin I. As illustrated in Figure 5, LTD induction for 60 min caused a significant increase in the phosphorylation levels of CaMKII (α and β) and of Synapsin I relative to the levels displayed by unstimulated slices (naïve). Slices pre-incubated for 1 h with 20 μM ryanodine before applying the LTD induction protocol displayed phosphorylation levels of CaMKII-α, CaMKII-β and Synapsin I that were not significantly different from the levels displayed by unstimulated slices (Figure 5). In addition, naïve slices pre-incubated for 1 h with 20 μM ryanodine displayed comparable CaMKII and Synapsin I phosphorylation levels as naïve control slices (Figure 5). The protein contents of CaMKII-α, CaMKII-β and Synapsin I were not affected by inhibitory ryanodine or exposure to the LTD induction protocol (Supplementary Figure S1).

**Figure 6:**
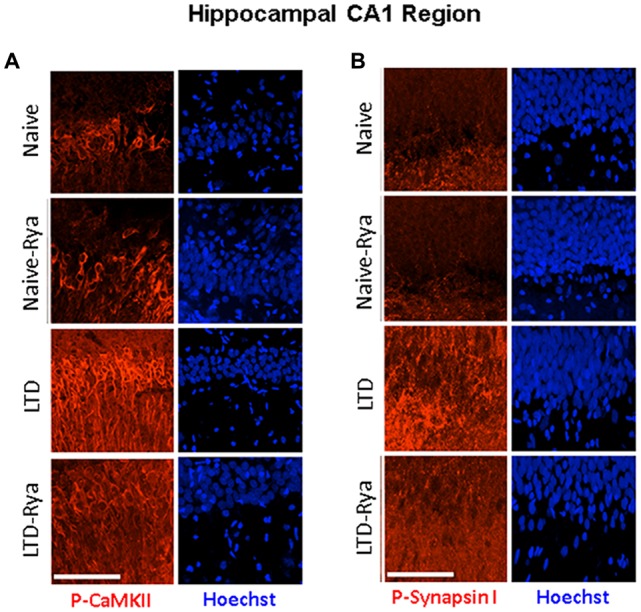
The LTD induction protocol increased **(A)** CaMKII and **(B)** Synapsin I phosphorylation levels in hippocampal sections. The figure illustrates representative immunohistochemistry images. **(A)** CA1-containing hippocampal section stained with an antibody against phospho-CaMKII or **(B)** with an antibody against phospho-Synapsin-I, which detects CaMKII-dependent phosphorylation of phospho-Ser-603. Three independent experiments yielded similar results. For experimental details, see text. The calibration bar represents 50 μm.

In agreement with these findings, immunohistochemistry analysis of hippocampal sections containing the CA1 region showed that LTD induction increased CaMKII phosphorylation relative to naïve slices in the soma of CA1 neurons and in neurite projections (Figure 6A). LTD induction also increased CaMKII-dependent Synapsin I phosphorylation in neurite projections; inhibitory ryanodine markedly reduced both increments (Figure 6B).

## Discussion

### Summary of Results

The results presented in this work show that treatment of hippocampal slices with concentrations of ryanodine (1 μM) or caffeine (1 mM) that are known to stimulate RyR channel activity did not change the parameters of the fEPSP waveforms in the synaptic response. Inhibitory ryanodine (20 μM), however, produced a modest increase in the half-width and the tau decay values of fEPSPs, but this response was observed only when applying strong stimulation. In addition, low or high concentrations of ryanodine, as well as 1 mM caffeine, did not modify basal synaptic transmission or PP responses. Treatment with 1 μM ryanodine or 1 mM caffeine enhanced LTD induction, while inhibitory ryanodine prevented the induction of LTD and decreased CaMKII and Synapsin I phosphorylation relative to the levels exhibited by control slices 1 h after LTD induction.

### Ryanodine and Caffeine Do Not Affect Fiber Volley Amplitude and Basal fEPSP Properties

The negative deflection of FV records evoked by extracellular stimulation represents the extracellular counterpart of action potentials in the presynaptic fibers (Henze et al., 2000). In our recordings, we found that 1 μM or 20 μM ryanodine, or 1 mM caffeine did not modify FV amplitude, suggesting that these drugs do not affect presynaptic components involved in basal synaptic transmission. These results add novel findings to the characterization of this presynaptic response.

We observed that both ryanodine and caffeine did not affect basal fEPSP amplitudes and slopes, in agreement with the previously reported lack of effect of 0.2 μM ryanodine on these fEPSP properties (Grigoryan et al., 2012). Based on these combined results, we suggest that RyR-mediated Ca^2+^ signals do not participate in modulating basal α-amino-3-hydroxy-5-methyl-4-isoxazolepropionic acid (AMPA) or NMDAR kinetics. A previous report, however, showed that more prolonged incubation (30 min) with a higher caffeine concentration (5 mM) modifies fEPSP slopes recorded in the ventral hippocampus but does not alter fEPSP slopes recorded in the dorsal hippocampus (Grigoryan et al., 2012), suggesting different responses to RyR activation by caffeine in these two regions. In our experiments, we did not differentiate between ventral and dorsal hippocampus and only tested the effects of incubation for 15 min with 1 mM caffeine.

In addition, albeit 1 μM ryanodine or caffeine did not modify the half-width times or the exponential decay constant tau, we found that inhibitory ryanodine modified these two parameters to a small extent but only under strong stimulation conditions. Possibly, strong stimulation enhances the emergence of RyR-mediated Ca^2+^ signals that promote repolarization by recruiting postsynaptic Ca^2+^-gated K^+^ channels. The hippocampus expresses both large-conductance (BK) and low conductance (SK) Ca^2+^-activated K^+^ channels (Chen et al., 2014). The BK channels are expressed in presynaptic terminals of Schaffer collateral fibers of *stratum radiatum* but do not contribute significantly to transmitter release under basal experimental conditions (Hu et al., 2001). In contrast, SK channels are expressed in hippocampal CA1 pyramidal cells (Stocker and Pedarzani, 2000; Bond et al., 2004; Chen et al., 2014). Moreover, SK2 is the most abundant isoform expressed in dendritic spines of CA1 pyramidal cells (Chen et al., 2014); this isoform is activated by Ca^2+^ entry through NMDARs and voltage-gated Ca^2+^ channels (Chen et al., 2014), and possibly by Ca^2+^ generated by RyR-mediated CICR from the ER in response to strong stimulation. We suggest, accordingly, that RyR channel inhibition decreases the activity of postsynaptic SK channels, causing the small increases of tau values and half-width times produced in response to strong stimulation.

### Ryanodine or Caffeine Do Not Affect Paired-Pulse Responses

We show in the present work that low or high concentrations of ryanodine or 1 mM caffeine did not modify the paired-pulse facilitation response, in agreement with a previous report showing that incubation for 30 min with 1 mM caffeine does not modify this response (Grigoryan et al., 2012). We add to these findings the lack of effect of low or high concentrations of ryanodine. We propose that RyR-mediated Ca^2+^ release does not participate in the fast-presynaptic neurotransmitter release that underlies pair pulse facilitation, which occurs in the tens of ms time range, but is required to stimulate slower Ca^2+^-dependent pathways underlying LTD induction, as discussed below.

### Ryanodine and Caffeine Modify LTD Induction

Previous reports have implicated Ca^2+^ release from the ER in the induction of LTD in young (Mulkey and Malenka, 1992; Reyes and Stanton, 1996; Nakano et al., 2004) and aged rodents (Kumar and Foster, 2005). In particular, thapsigargin and cyclopiazonic acid, two sarco/ER Ca^2+^-ATPase inhibitors that deplete intracellular Ca^2+^ stores (Seidler et al., 1989; Thastrup et al., 1990), inhibit LTD induction by 1 Hz stimulation in the hippocampal CA3–CA1 synapse in young rats (Reyes and Stanton, 1996). A later report, however, described that LTD induction by 1 Hz stimulation was not affected by thapsigargin and cyclopiazonic acid; yet, 0.5 or 2 Hz stimulation, which induced much smaller LTD, required Ca^2+^ stores (Nakano et al., 2004).

A few studies have implicated IP_3_R channels in LTD induction in CA1 neurons of guinea pig hippocampal slices (Taufiq et al., 2005), in postsynaptic cerebellar Purkinje neurons (Finch and Augustine, 1998) and in Schaffer collateral-CA1 synapses (Jo et al., 2010). Alternatively, other reports have implicated RyR channels in LTD induction in young (Reyes and Stanton, 1996; Nakano et al., 2004) and aged rodents (Kumar and Foster, 2005). Moreover, the RyR3 channel isoform was implicated in LTD induction, based on the finding that one train of LFS does not induce LTD in RyR3-deficient mice (Futatsugi et al., 1999). Here, we present additional results that confirm the role of RyR channels in LTD induction by 1 Hz, by showing that stimulatory ryanodine and caffeine enhanced LTD induction while inhibitory ryanodine abolished this response.

In addition to RyR channels, several neuronal sources contribute to elicit the postsynaptic Ca^2+^ signals required for LTD induction in response to LFS, including NMDARs (Dudek and Bear, 1992; Bear and Abraham, 1996; Li et al., 2007), AMPA receptors (Sanderson et al., 2016) and metabotropic receptors (Taufiq et al., 2005; Citri and Malenka, 2008; Hisatsune and Mikoshiba, 2017). As mentioned above, a role for IP_3_R channels in LTD induction in different synapse types has been reported (Finch and Augustine, 1998; Taufiq et al., 2005; Jo et al., 2010). Yet, Ca^2+^ release mediated by IP_3_R1 channels, which undergo activation via G-protein-coupled metabotropic glutamate receptors (Hisatsune and Mikoshiba, 2017), is unlikely to participate in LTD induction. Mice lacking the IP_3_R1 type-1 (IP_3_R1) isoform, the more abundant IP_3_R isoform expressed in the hippocampus (Furuichi et al., 1994; Hertle and Yeckel, 2007; Hisatsune and Mikoshiba, 2017), display robust LTD induction in response to LFS protocols (Fujii et al., 2000; Nagase et al., 2003).

### Inhibitory Ryanodine Reduces the Enhanced Phosphorylation of CaMKII and Synapsin I Caused by LTD Induction

We found that 1 h after LTD induction slices exhibited a significant increase in CaMKII (α and β) and Synapsin I phosphorylation; these increments did not occur in slices treated with inhibitory ryanodine prior to exposure to the LTD induction protocol. In addition, LTD induction enhanced RyR-mediated Synapsin I phosphorylation by CaMKII, which we interpret as evidence of presynaptic CaMKII activation following LTD induction. The elimination by inhibitory ryanodine of the enhanced CaMKII-dependent Synapsin I phosphorylation indicates RyR-mediated Ca^2+^ signals mediate the LTD-induced stimulation of presynaptic CaMKII activity. Based on these novel results, we suggest that LTD induction requires presynaptic functional RyR channels to generate Ca^2+^ signals that stimulate CaMKII activity in presynaptic terminals.

In addition, we found that inhibitory ryanodine partially prevented the enhanced CaMKII phosphorylation displayed by the soma of CA1 neurons 1 h after LTD induction. Whether this postsynaptic increase is relevant for LTD induction remains the subject of future studies, albeit an earlier report showed that LTD induction requires presynaptic but not postsynaptic CaMKII activation (Stanton and Gage, 1996).

Several studies indicate that Synapsin I phosphorylation, which displayed a significant increase following LTD induction (Figures 5, 6), leads to an increase in the readily releasable vesicle pool and enhances vesicle availability for exocytosis (Chi et al., 2003). Yet, studies in Synapsin knockout mice suggest that CaMKII-mediated Synapsin I phosphorylation has a limited role in controlling neurotransmitter release (Gitler et al., 2008; Wang, 2008; Song and Augustine, 2015). To our knowledge, there is no information in the literature implicating Synapsin I phosphorylation as part of the cellular mechanisms underlying LTD induction, which for NMDA-dependent LTD at CA3–CA1 synapses entail long-term reduction of release from the rapidly recycling presynaptic vesicle pool (Zhang et al., 2006).

## Conclusion

Based on the present findings, we propose that the Ca^2+^ signals generated by activation of presynaptic RyR channels enhance LTD induction while their inhibition precludes this response. Accordingly, we suggest that RyR-mediated Ca^2+^ release from presynaptic intracellular stores contributes to the activation of downstream Ca^2+^-dependent pathways and signaling molecules, including CaMKII, which are required for LTD induction.

## Author Contributions

AA-C designed and performed all experiments, analyzed results, wrote the first draft of the manuscript and revised all subsequent version of the manuscript. GB analyzed results, and revised all versions of the manuscript. GS performed immunoblots, analyzed the ensuing results and contributed to manuscript writing. CE provided the software for analysis of results. PM performed electrophysiological and immunohistochemistry experiments, analyzed the results generated by them and contributed to manuscript writing. CH supervised and financed the work, analyzed results and wrote the final version of the manuscript.

## Conflict of Interest Statement

The authors declare that the research was conducted in the absence of any commercial or financial relationships that could be construed as a potential conflict of interest.

## Supplementary Material

FIGURE S1 The LTD induction protocol does not modify hippocampal CaMKII and Synapsin I protein contents. The protein contents of CaMKII-alpha (left panel), CaMKII-beta (center panel) and Synapsin I (right panel), measured 1 h after applying the LTD induction protocol to control (C) or to slices treated with inhibitory ryanodine (Rya), were not significantly different from the respective protein contents of naïve slices. Values represent Mean ± SE (*n* = 3). Statistical analysis was performed with one-way ANOVA, followed by Tukey’s *post hoc* test.

## Abbreviations

ACSF: artificial cerebrospinal fluid
AMPA: α-amino-3-hydroxy-5-methyl-4-isoxazolepropionic acid
BK: large-conductance Ca^2+^-activated K^+^ channels
CICR: Ca^2+^-induced Ca^2+^ release
ER: endoplasmic reticulum
fEPSP: field excitatory postsynaptic potential
FV: fiber volley
I/O: input/output
IP_3_: inositol 1,4,5-trisphosphate
IP_3_R: IP_3_ receptor
LFS: low frequency stimulation
LTD: long-term depression
LTP: long-term potentiation
NMDA: *N*-methyl-D-aspartate
PP: Paired Pulse
RyR: ryanodine receptor
SK: low-conductance Ca^2+^-activated K^+^ channels
TBS: theta-burst stimulation.
