# Supplementary figures and images for: Ryanodine Receptor-Mediated Calcium Release Has a Key Role in Hippocampal LTD Induction
Source: Front Cell Neurosci. 2018 Nov 6;12:403. doi: 10.3389/fncel.2018.00403 (PMC6232521; doi:10.3389/fncel.2018.00403)

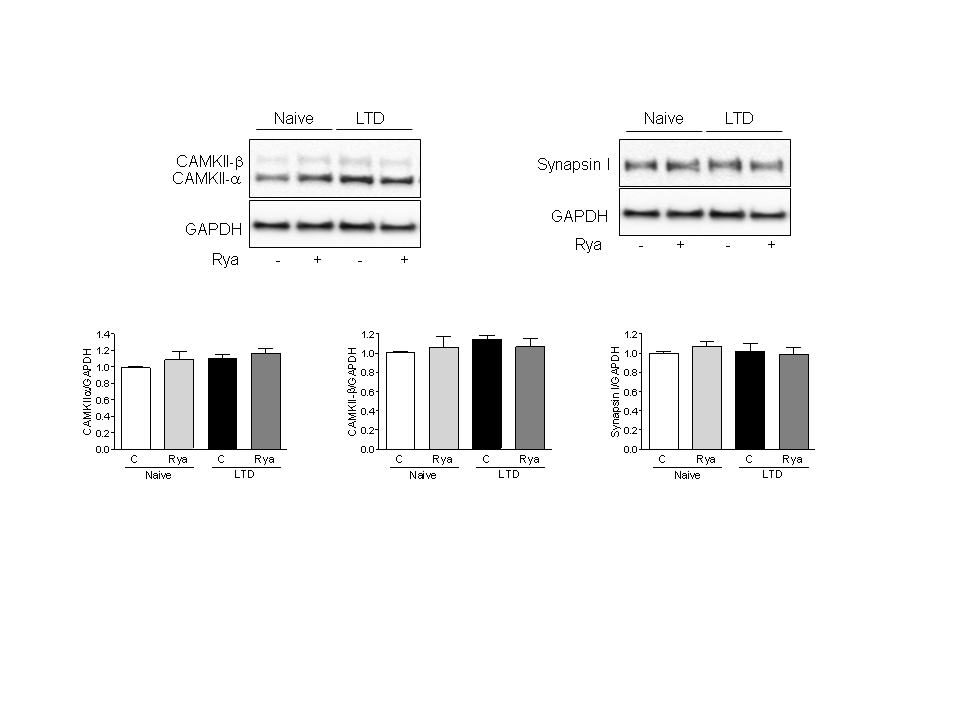

Supplement: FIGURE S1 — The LTD induction protocol does not modify hippocampal CaMKII and Synapsin I protein contents. The protein contents of CaMKII-alpha (left panel), CaMKII-beta (center panel) and Synapsin I (right panel), measured 1 h after applying the LTD induction protocol to control (C) or to slices treated with inhibitory ryanodine (Rya), were not significantly different from the respective protein contents of naïve slices. Values represent Mean ± SE (n = 3). Statistical analysis was performed with one-way ANOVA, followed by Tukey’s post hoc test. [file Image_1.TIF]
